# Skeletal muscle AMPK is essential for the maintenance of FNDC5 expression

**DOI:** 10.14814/phy2.12343

**Published:** 2015-05-06

**Authors:** James S V Lally, Rebecca J Ford, Jasper Johar, Justin D Crane, Bruce E Kemp, Gregory R Steinberg

**Affiliations:** 1 Division of Endocrinology and Metabolism, Department of Medicine, McMaster UniversityHamilton, Ontario, Canada; 2 St. Vincent's Institute of Medical Research and Department of Medicine, University of MelbourneFitzroy, Victoria, Australia; 3 Department of Biochemistry and Biomedical Sciences, McMaster UniversityHamilton, Ontario, Canada

**Keywords:** AMPK, exercise, FNDC5, skeletal muscle

## Abstract

Fibronectin type III domain-containing protein 5 (FNDC5) expression is controlled by the transcriptional co-activator, peroxisome proliferator-activated receptor gamma, coactivator 1 alpha (PGC1α). FNDC5 expression has been shown to be increased in muscle in response to endurance exercise in some but not all studies, therefore a greater understanding of the mechanisms controlling this process are needed. The AMP-activated protein kinase (AMPK) is activated by exercise in an intensity dependent manner and is an important regulator of PGC1α activity; therefore, we explored the role of AMPK in the regulation of FNDC5 using AMPK β1β2 double muscle-null mice (AMPK DMKO), which lack skeletal muscle AMPK activity. We found that FNDC5 expression is dramatically reduced in resting muscles of AMPK DMKO mice compared to wild-type littermates. In wild-type mice, activating phosphorylation of AMPK was elevated immediately post contraction and was abolished in muscle from AMPK DMKO mice. In contrast, PGC1α was increased in both wild-type and AMPK DMKO mice 3 h post contraction but FNDC5 protein expression was not altered. Lastly, acute or chronic activation of AMPK with the pharmacological AMPK activator AICAR did not increase PGC1α or FNDC5 expression in muscle. These data indicate that skeletal muscle AMPK is required for the maintenance of basal FNDC5 expression.

## Introduction

The health benefits of regular exercise are undisputed but the molecular mechanisms mediating the effects remain unclear. It has been suggested that secreted factors from skeletal muscle (myokines) may modulate some of the beneficial health effects of exercise (Pedersen and Febbraio 2012). Recently a novel myokine encoded by the fibronectin type III domain-containing protein 5 (FNDC5) gene that gives rise to a released fragment (amino acids 29–140) termed irisin, was found to be produced in response to peroxisome proliferator-activated receptor γ co-activator-1α (PGC-1α) activation following exercise training (Bostrom et al. 2012). A number of papers have begun to provide valuable data on FNDC5/irisin including its structure (Schumacher et al. 2013), signaling in white adipose tissue (Zhang et al. 2014), participation in the adaptation to cold, (Lee et al. 2014), and importance in regulating brain derived brain-derived neurtrophic factor (BDNF) production in the hippocampus (Wrann et al. 2013). Unfortunately, initial exuberance surrounding irisin has led to an explosion of clinically based studies that rely heavily the ability to detect irisin in circulation, which now appears questionable (Albrecht et al. 2015) and have revealed few mechanistic insights. How exercise mediates changes in FNDC5 expression is not fully understood, and notably irisin expression has not been observed during all types of exercise, especially in humans (Pekkala et al. 2013; Raschke et al. 2013). Therefore, a greater understanding of factors controlling FNDC5/irisin expression is warranted.

AMP-activated protein kinase (AMPK) is a multi-substrate kinase that is activated in skeletal muscle during exercise (Steinberg and Jorgensen 2007; Richter and Ruderman 2009). AMPK exists as a heterotrimeric protein composed of α (α_1_ & α_2_), β (β_1_ & β_2_) and γ (γ_1_, γ_2_ & γ_3_) subunits (Steinberg and Kemp 2009). AMPK activation is linked to PGC-1α function, as AMPK can directly phosphorylate PGC1α, a process that is important for auto-regulatory mechanism by which PGC-1α perpetuates its own expression (Jäger et al. 2007; O'Neill et al. 2013). We have recently generated mice lacking muscle AMPK activity through targeted deletion of both AMPK β1 and β2 subunits (O'Neill et al. 2011). AMPK double β muscle knock-out mice (AMPK DMKO) have no detectable muscle AMPK activity and are characterized by dramatic reductions in exercise capacity and muscle mitochondrial content. Given the intimate connection between endurance exercise, AMPK and PGC1α activity we hypothesized that FNDC5 may be reduced in AMPK DMKO mice. We find that muscle FNDC5 expression is lower in muscle of AMPK DMKO mice.

## Materials and Methods

### Animals

AMPK DMKO mice have been described previously (O'Neill et al. 2011). AMPK DMKO and wild-type littermates were housed in specific pathogen free micro-isolator cages maintained under a 12 h light/dark cycle at a constant temperature of 23°C. Mice had access to standard chow and water ad libitum. All of the experiments performed in this study were approved by the McMaster University Animal Research Ethics Board.

### Treadmill exercise

Before the treadmill running experiments, mice were allowed to acclimatize to the treadmill apparatus as described previously (O'Neill et al. 2011). Maximal running speed was established by allowing the mice to run at 10 m/min for 2 min with an incline of 5 degrees. The speed of the treadmill was then increased by 1 m/min every 2 min until mice could not be prompted to continue running. The following week mice underwent an acute, submaximal bout of exercise. Mice were run at 10 m/min for two minutes, followed by an increase in the treadmill speed to 70% of maximal running velocity until volitional exhaustion.

### In situ contraction experiments

In situ contraction experiments were performed as we have described previously (Dzamko et al. 2008), with some modifications. Mice were anesthetized using inhalation anesthetic (1–2% isoflurane in O_2_), the tibialis anterior muscle was surgically exposed, the distal tendon secured using a piece of 5.0 suture and severed from its attachment point. Mice were mounted to an 809B in situ Mouse Apparatus platform (Aurora Scientific Inc., Aurora, ON, Canada) maintained at constant temperature of 33°C. Using the free end of the suture, the tendon was attached to a 300C dual mode muscle lever system (Aurora Scientific) and the muscle was stimulated to contract via two small platinum hook electrodes place around sciatic nerve. Optimal muscle length was determined by administering single electrical pulses (200 ms at 2–3 mA) while lengthening the muscle until optimal twitch force was attained. The muscle was stimulated 400 times at 10 hz for 0.5 sec with 3.7 sec rest between contractions. The amperage was adjusted between 1.5 and 4.0 mA to maintain a target force of 250 mN. Upon completion, the tibialis anterior muscle was excised and flash frozen in liquid nitrogen either immediately or 3 h post contraction and stored at −80°C for subsequent molecular analysis.

### AICAR administration

Mice received a daily injection of saline (control) or AICAR (500 mg/kg) for a 2-week period. Blood glucose was measured on whole blood using a handheld glucometer one hour after the first injection. One hour following the final injection the mice were anesthetized using ketamine/xylanzine as before, tissues were collected and flash frozen using liquid nitrogen and stored at −80°C for subsequent molecular analysis.

### Protein and mRNA analysis

For real time PCR analysis, total RNA was extracted from 8 to 20 mg of muscle tissue using a combination Trizol reagent (Life Technologies Inc., Burlington, ON, Canada) and RNeasy mini kit (Qiagen, Toronto, ON, Canada). cDNA was generated from 0.5 μg of total RNA with Superscript III reverse transcriptase (Invitrogen) using random primers. The levels of *Fndc5*,* Pgc1α* and *Tbp* (TATA-binding protein) were determined using TaqMan assays on demand (Invitrogen). Relative RNA abundance was calculated using the ΔΔ*C*_T_ method as we have described previously (O'Neill et al. 2011) using *Tbp* as an endogenous control.

Protein abundance was determined using Western blot analysis as we have described previously (O'Neill et al. 2011) using the following commercially available antibodies: Acetyl-CoA Carboxylase (ACC) (#3676), ACC pSer79 (#3661), AMPK pan α (#2532), AMPK pThr172 (#2532), GAPDH (#5174), all from Cell Signaling Technology, Inc., Danvers, MA and FNDC5 using Irisin (42–112) purified IgG antibody (Phoenix Pharmaceuticals, Burlingame, CA).

### Statistics

All data are expressed as means plus or minus the SEM Statistical analysis was carried out using Graphpad Prism6 software; GraphPad Software, Inc., La Jolla, CA. Student's *t*-test or two-way ANOVA with Fisher's LSD post-hoc test were used where appropriate. Significant differences were reported if *P *≤* *0.05.

## Results

### FNDC5 expression is lower in AMPK DMKO muscle and is not affected by exercise or muscle contraction

**Figure 1:**
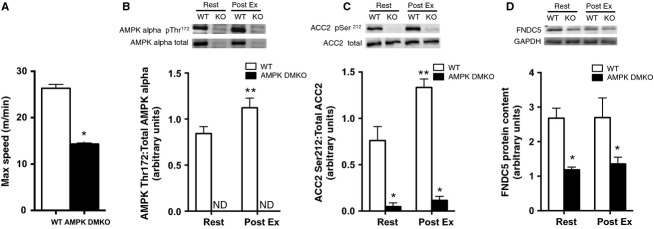
Expression of FNDC5 is lower in muscle tissue from AMPK DMKO mice compared to wild-type mice but is unaltered after exhaustive treadmill exercise. (A) The maximum speed attained during a progressive treadmill running exercise capacity test in wild-type and AMPK DMKO mice. AMPK alpha pThr172 (B) and ACC2 pSer212 (C) phosphorylation levels and FNDC5 protein content (D) at rest (Rest) or immediately following exercise (Post Ex) in tibialis anterior muscle tissue (*N* = 3–5). Error bars represent standard error of the mean. ND, not detectable. *Significantly different from wild-type, *P* < 0.05. **Significantly different from wild-type Rest.

We examined whether an acute bout of treadmill exercise can affect FNDC5 expression and whether AMPK might be important. A progressive treadmill exercise test was used to establish the maximum running speed of wild-type and AMPK DMKO mice. In line with previous findings (O'Neill et al. 2011), AMPK DMKO were severely exercise intolerant (wild-type = 26 m/min vs AMPK DMKO = 14 m/min, *P* < 0.0001, Fig.1A). The following week mice were either rested or performed a single bout of exhaustive exercise at 70% of their relative maximum speed (18 m/min for wild-type mice vs. 10 m/min for AMPK DMKO), which was sufficient to activate AMPK and phosphorylate its downstream substrate ACC at the Ser212 site in skeletal muscle tissue from wild-type but not AMPK DMKO mice (Fig.1B and C). We found that FNDC5 protein expression was reduced in AMPK DMKO mice both at rest and following treadmill exercise (Fig.1D). Despite robust AMPK activation there was no increase in skeletal muscle FNDC5 protein levels immediately following exercise in wild-type mice (Fig.1D). These data indicate that AMPK is important for controlling basal/resting muscle FNDC5 expression but a single bout of treadmill exercise that activates AMPK is insufficient to increase FNDC5 protein expression.

**Figure 2:**
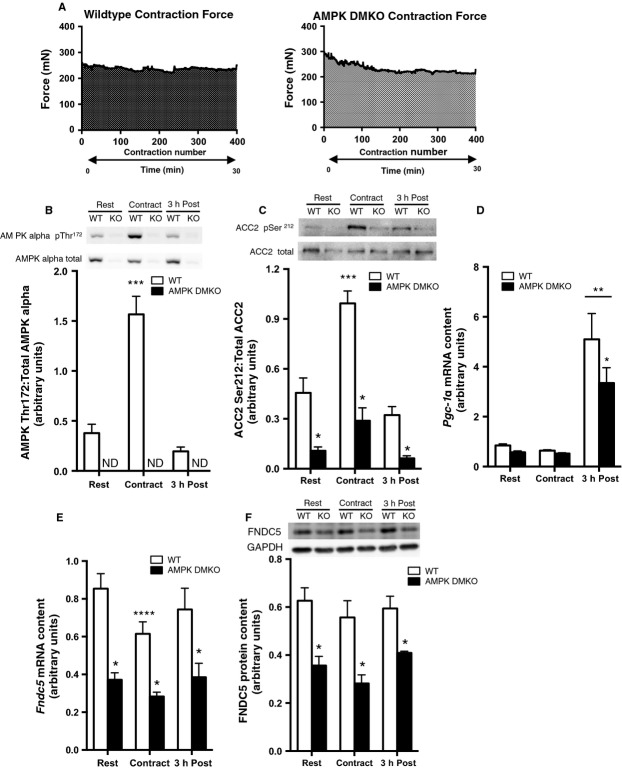
FNDC5 expression in muscle from wild-type and AMPK DMKO mice at rest and after force-matched in situ contraction. (A) Tibialis anterior force generation over 400 contractions conducted over 30 min in wild-type (left) and AMPK DMKO mice (right). AMPK alpha pThr172 (B) and ACC2 pSer212 (C) phosphorylation levels, *Pgc1α* (D) and *Fndc5* mRNA (E) and FNDC5 protein (F) content at Rest, immediately following (Contract), and 3 h post contraction (3 h Post) in the tibialis anterior muscle of wild-type and AMPK DMKO mice (*N* = 3–5). Error bars represent standard error of the mean. ND, not detectable. *Significantly different from wild-type, *P* < 0.05. **Significantly different from Rest and Contract, *P* < 0.05. ***Significantly different from Rest and 3 h Post, *P* < 0.05. ****Significantly different from wild-type Rest, *P* < 0.05.

We next developed an in situ approach that allows for the precise measurement of the force of each contraction, while maintaining blood flow and neural inputs to contracting muscle. The tibialis anterior muscle was stimulated to contract 400 times and the force output was precisely matched between wild-type and AMPK DMKO mice over approximately 30 min (Fig.2A). Muscles were collected at rest, immediately post contraction or they were allowed to recover in situ for 3 h after completion of the contraction series.

In wild-type mice AMPKα activating Thr172 phosphorylation was increased immediately post-contraction (4-fold, *P* < 0.001, Fig.2B) and had returned to basal levels 3 h later. AMPK DMKO mice had no detectable AMPK Thr172 phosphorylation at any time point consistent with our previous observations indicating the lack of skeletal muscle AMPK activity in this mouse model (O'Neill et al. 2011). Phosphorylation of the AMPK substrate ACC2 at Ser 212 was elevated post contraction in wild-type but not in AMPK DMKO muscle tissue (Fig.2C). Importantly, the increase in ACC phosphorylation in WT mice was comparable to that elicited following treadmill running (Fig.1C). There was no difference in PGC1α expression between genotypes at rest or immediately post-contraction, however 3 h later PGC1α expression was increased in contracted tibialis anterior muscle from both wild-type and AMPK DMKO mice (6- to 8-fold, *P* < 0.001, Fig.2D). The increase was blunted in muscle from AMPK DMKO animals, indicating that AMPK independent pathways are important for controlling PGC1α expression during muscle contractions (Fig.2D). Despite the robust increases in *Pgc1α* mRNA three hours post contraction there was no increase in *Fndc5* mRNA or protein content (Fig.2E and F). Importantly, FNDC5 expression remained lower in AMPK DMKO mice before, immediately after and 3 h post muscle contractions (Fig.2E and F).

### Acute or chronic administration of AICAR does not affect FNDC5 expression

**Figure 3:**
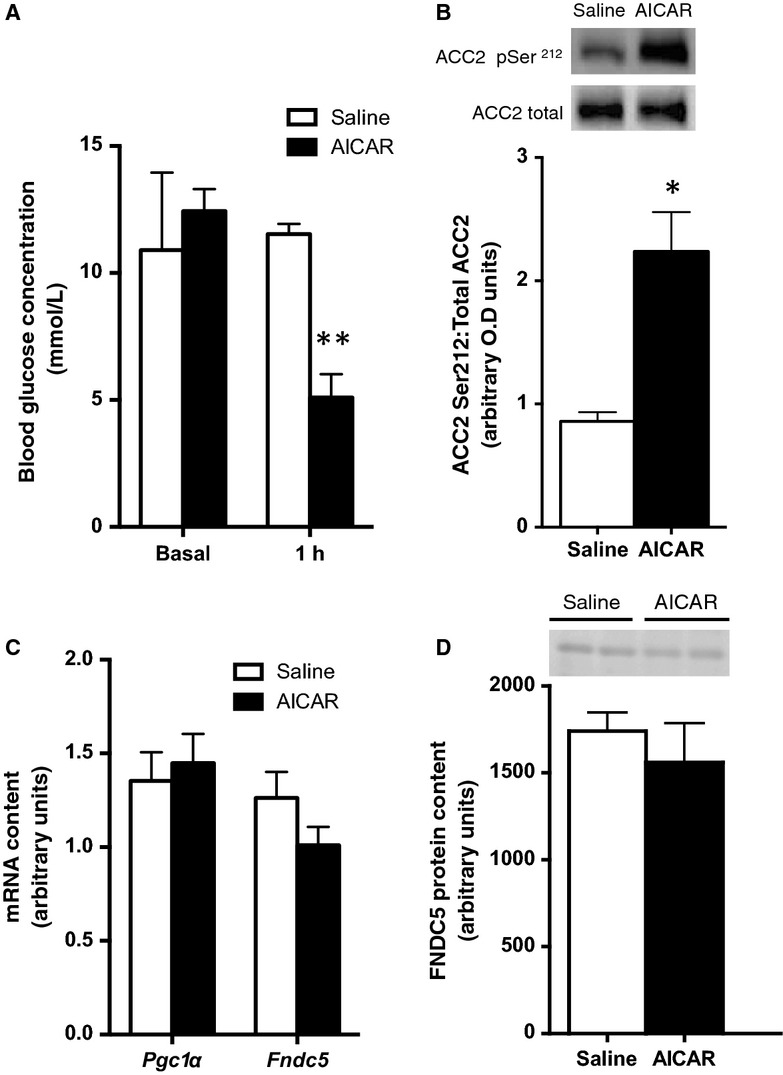
FNDC5 is not affected by acute or chronic AICAR treatment. Blood glucose (A) one hour after the administration of an acute I.P. injection of AICAR in wild-type mice (*N* = 6). ACC2 pSer212 (B) phosphorylation levels and *Pgc1α* and *Fndc5* mRNA (C) and FNDC5 protein content (D) in tibialis anterior muscle of mice chronically treated with AICAR for 2 weeks (*N* = 5). Error bars represent standard error of the mean. *Significantly different from saline control. **Significantly different from basal and 1 h saline, *P* < 0.05.

Given the regulation of FNDC5 expression by AMPK in resting but not contracting muscle we next tested whether pharmacological activation of AMPK using acute or chronic administration of the AMPK agonist 5-Aminoimidazole-4-carboxamide ribonucleotide (AICAR) could increase FNDC5 levels in wild-type mice. Acute AICAR injection increased AMPK phosphorylation of ACC2 at Ser212 (Fig.3A, 2.6-fold, *P* < 0.001) and lowered blood glucose after 1 h in agreement with previous studies (Steinberg et al. 2010; Fig.3B). Chronic daily administration of AICAR over a 2-week period did not alter *Pgc1α* or *Fndc5* mRNA content (Fig.3C) or FNDC5 protein levels (Fig.3D) in tibialis anterior muscle.

## Discussion

FNDC5/irisin was first characterized as a muscle secreted factor regulated by PGC1α that was increased in response to endurance exercise training. Despite extensive examinations of the metabolic functions of this novel protein few studies have examined the mechanisms by which acute exercise/muscle contractions increase FNDC5 expression. While several reports have questioned whether skeletal muscle FNDC5 expression is increased following exercise (Timmons et al. 2012; Pekkala et al. 2013; Raschke et al. 2013) there is emerging evidence that during high-intensity endurance exercise that FNDC5 expression is increased (Huh et al. 2014). However, the factors upstream of PGC1α that control FNDC5 expression in response to muscle contractions/exercise have yet to be delineated.

We found that muscle-specific deletion of AMPK lowered basal expression levels of FNDC5 in tibialis anterior muscle. Thus our data establish a novel linkage between skeletal muscle AMPK and the maintenance of basal FNDC5 expression. This may involve an AMPK-PGC1α-FNDC5 regulatory axis, as AMPK has been shown to act upstream of PGC1α (O'Neill et al. 2013). Previous work has also suggested a relationship between AMPK-PGC1α-FNDC5 as a consequence of whole-body myostatin deletion (Shan et al. 2013). This group showed that AMPK inhibition through treatment with compound C, elicited a decrease in FNDC5 expression in myostatin null myotubes. It is notable that compound C also inhibits more than 10 other protein kinases with greater or equal efficacy to AMPK and importantly many of these protein kinases are known regulators of PGC1α activity (Bain et al. 2007), therefore further studies confirming these effects in genetic models are warranted. One potential confounding factor when using transgenic models with alterations in major metabolic regulatory nodes such as AMPK is that the animals may have reduced activity levels. In such cases muscle disuse can drive phenotypic changes that cannot be solely attributed to alterations in AMPK. It is important to note that previous work from our laboratory has shown that AMPK DMKO do not have reduced activity levels (O'Neill et al. 2011) suggesting that the reduction in FNDC5 expression observed in AMPK DMKO muscle tissue is unlikely to be due to reduced physical activity. Ideally future studies will investigate whether an acute/inducible reduction in AMPK expression also lowers FNDC5 expression.

Skeletal muscle AMPK is activated in response to energetic stresses such as endurance exercise which disrupts the adenylate charge of the cell (Steinberg and Jorgensen 2007). Consistent with this idea AMPK is activated in an intensity dependent manner (Fujii et al. 2000; Wojtaszewski et al. 2000; Chen et al. 2003) and is essential for allowing animals to perform endurance treadmill running (O'Neill et al. 2011). Given the limitation of the treadmill exercise experiments as a result of the exercise intolerance of the AMPK DMKO mice we proceeded to do experiments under more controlled settings in which absolute contraction intensities could be matched between genotypes. Sustained and force-matched in situ contraction caused robust and expected AMPK activation and increases in *Pgc1α* expression in wild-type and AMPK DMKO mice. To our knowledge this is the first evidence that contraction-stimulated increases in *Pgc1α* occur in mice genetically lacking both AMPK subunits. The robust elevation in *Pgc1α* mRNA levels post-exercise is well documented (Hildebrandt et al. 2003), and temporal and intensity related changes occur in both rodents (Tadaishi et al. 2011) and humans (Mathai et al. 2008; Perry et al. 2010). Importantly, this occurred after a matched bout of contraction sufficient to activate AMPK in wild-type murine muscle, indicating that AMPK is required for full PGC1α induction, at least at modest intensities; an idea consistent with the notion that AMPK mediates PGC1α autoregulation (Handschin et al. 2003).

Interestingly, despite this robust increase in *Pgc1α* expression there was no change in FNDC5 mRNA or protein expression in skeletal muscle following in situ contractions. The reason for the dichotomy between *Pgc1α* and FNDC5 expression in response to muscle contractions is not known. However it appears not to be related to the more modest increase in AMPK activity, as ACC phosphorylation was comparable in wild-type mice following either treadmill exercise (Fig.2C) or in situ muscle contraction (Fig.3C). A number of other upstream kinases have been implicated in regulating PGC1α activity including p38 mitogen activated protein kinase (Puigserver et al. 2001; Akimoto et al. 2005) and Ca^2+^/calmodulin-dependent protein kinase II (Wright et al. 2007). It is interesting to speculate that the exhaustive treadmill exercise elicits other signals, which are required to synergize with PGC1α to elicit FNDC5 expression and are not present during the in situ muscle contraction. It is also possible that the intensity of contraction used to match force between wild-type and AMPK DMKO muscle in situ was too low to drive robust changes in FNDC5 expression. Our data demonstrate that the contraction intensity achieved in our in situ system was sufficient to activate AMPK in wild-type muscle while driving PGC1α expression in both wild-type and AMPK deficient muscle. Despite this substantial activation of the AMPK-PGC1α axis there was no change in FNDC5 expression suggesting that other factors may be required to increase FNDC5 expression during muscle contractions.

Given the complexities of matching exercise intensities and muscle force between wild-type and AMPK DMKO mice we conducted studies with AICAR, which increases skeletal muscle AMPK activity (Winder and Hardie 1996) and has shown to increase PGC1α expression via an AMPKα2 and PGC1α dependent pathway (Jorgensen et al. 2005). We conducted 2 weeks of daily AICAR treatment and although AICAR elicited the expected phosphorylation of ACC2 and reduced blood glucose levels consistent with the activation of skeletal muscle AMPK that we (Dzamko et al. 2008; Steinberg et al. 2010) and others (Barnes et al. 2004; Jorgensen et al. 2004) have observed, it failed to increase FNDC5 expression. Therefore, chronic activation of AMPK via AICAR administration is not sufficient to cause changes in FNDC5 expression in vivo.

## Conclusions

This study is the first to find that skeletal muscle AMPK is required to maintain basal expression of FNDC5 in skeletal muscle. Neither an acute bout of exhaustive exercise nor force-matched in situ muscle contractions, that induce comparable increases in AMPK activity, resulted in increases in FNDC5 expression in either wild-type or AMPK DMKO mice. Lastly, chronic pharmacological activation of AMPK using AICAR is insufficient to increase FNDC5 skeletal muscle. These studies indicate that AMPK is essential for maintaining basal FNDC5 expression but that other factors may contribute to increases in FNDC5 during exercise/muscle contractions.

## Conflict of Interest

None declared.
